# Effectiveness of Self-Management of Blood Glucose in Improving Glycemic Control in Patients With Diabetes: A Systematic Review and Meta-Analysis

**DOI:** 10.7759/cureus.99751

**Published:** 2025-12-21

**Authors:** Mohammed F Alserhani, Faisal Alrasheed, Abdullah M Al Qamshah, Yasser Alkharashi, Meshal F Aljarallah, Faisal F Alarifi, Eyad Althaqeb, Mansour I Alrasheed, Leena M Alkharashi, Eidan Al Eidan

**Affiliations:** 1 Family Medicine, Ministry of National Guard Health Affairs, Riyadh, SAU; 2 College of Medicine, King Saud Bin Abdulaziz University for Health Sciences, Riyadh, SAU; 3 Family Medicine and Primary Care, King Abdulaziz Medical City, Riyadh, SAU; 4 Family Medicine and Polyclinic, King Faisal Specialist Hospital and Research Centre, Riyadh, SAU; 5 College of Medicine, Almaarefa University, Riyadh, SAU; 6 King Abdullah International Medical Research Centre, King Abdulaziz Medical City, Riyadh, SAU

**Keywords:** diabetes mellitus, diabetes type 2, diabetic patients, glycated hemoglobin (hba1c), glycemic control, self-monitoring of blood glucose (smbg)

## Abstract

Self-monitoring of blood glucose (SMBG) is a widely used strategy in diabetes management, allowing patients to track their glucose levels and make informed decisions regarding diet, medication, and lifestyle adjustments. The effectiveness of SMBG remains debated. Structured SMBG, which involves systematic monitoring with clear guidance, has been suggested to provide greater benefits compared to unstructured SMBG. We aimed to evaluate the effectiveness of SMBG in improving glycemic control among diabetic patients.

A systematic search was conducted in PubMed, the Cochrane Library, and Google Scholar. Studies were included if they examined SMBG interventions and reported glycated hemoglobin (HbA1c) as an outcome. Randomized controlled trials (RCTs) and observational studies were assessed for quality using the Cochrane Risk of Bias Tool and the Newcastle-Ottawa Scale. Meta-analysis was performed using Review Manager (RevMan, The Cochrane Collaboration, Copenhagen, Denmark) to calculate mean differences (MD) and 95% confidence intervals (CI).

The comprehensive database search yielded 7,667 records, of which 22 articles were selected for review and analysis. The meta-analysis of 22 studies showed that SMBG significantly reduced HbA1c levels compared to no SMBG (MD = -0.32%, 95% CI: -0.44% to -0.20%). Structured SMBG resulted in a greater reduction (MD = -0.25%, 95% CI: -0.41% to -0.09%) compared to unstructured SMBG. In summary, we showed that SMBG is effective in lowering HbA1c, particularly when structured protocols are followed. Healthcare providers should promote structured SMBG, along with patient education, to enhance adherence and optimize glycemic control. Further long-term studies are necessary to evaluate long-term benefits.

## Introduction and background

Diabetes mellitus is a chronic metabolic disorder characterized by persistent hyperglycemia resulting from insulin resistance, reduced insulin secretion, or both. Achieving reasonable glycemic control is essential to prevent complications, including retinopathy, nephropathy, neuropathy, and cardiovascular diseases [1]. Self-monitoring of blood glucose (SMBG) is one of the many diabetes management strategies and has been shown to be a useful way for patients to determine their blood glucose levels in real time, enabling them to make informed decisions about diet, physical activity, and medication [2,3]. The American Diabetes Association guidelines and other clinical organizations recommend SMBG, especially for insulin-treated patients; however, its effectiveness for non-insulin-treated patients has been debated [4].

Several studies have examined the effects of SMBG on glycemic control, yielding varying results. Research indicates that SMBG is beneficial in reducing glycated hemoglobin (HbA1c) levels. SMBG can be classified as either structured or unstructured. Structured SMBG involves predefined testing schedules, with clear guidance on how results should be interpreted and management adjusted in response to the findings [5]. In contrast, unstructured SMBG lacks consistency and structured feedback. Structured SMBG has also resulted in significant decreases in HbA1c among non-insulin-treated patients with type 2 diabetes [5]. Similarly, Xu et al. [6] found that improved glucose control was associated with a higher frequency of SMBG. Moreover, Bosi et al. [7] found that intensive SMBG interventions achieved better glycemic outcomes. However, not all studies confirm these benefits. Parsons et al. [8] found that, in non-insulin-treated patients with type 2 diabetes, noninterventional SMBG did not result in a significant HbA1c reduction compared to patients who did not implement SMBG. Noting that SMBG provided valuable glucose data but did not translate into meaningful glycemic improvements in the absence of structured educational interventions, Parkin et al. also stated that SMBG offered a separate, critical function in reducing the risk of hypoglycemia [9]. This conflicting evidence raises questions about when SMBG is most beneficial.

To reconcile these inconsistencies, this systematic review and meta-analysis aimed to investigate the role of SMBG in glycemic control among diabetic patients. The distinction between structured and unstructured SMBG is also essential to consider. Additionally, this study will investigate variables that impact the effectiveness of SMBG. Variation in adherence to SMBG is substantial due to barriers such as financial constraints and lack of motivation [10].

## Review

Materials and methods

Study Design and Sources

This systematic review and meta-analysis adheres to the Preferred Reporting Items for Systematic Reviews and Meta-Analyses (PRISMA) guidelines [11]. The purpose was to evaluate whether the use of self-monitoring of blood glucose (SMBG) helps improve glycemic control in patients with diabetes. Randomized controlled trials (RCTs) and observational studies that reported glycated hemoglobin (HbA1c) outcomes were included in the review. A systematic literature search was conducted in PubMed, the Cochrane Library, and Google Scholar to provide a comprehensive synthesis of available evidence. Quantitative synthesis was then conducted on studies that met predefined eligibility criteria.

Literature Search Strategy

The structured search strategy utilized Medical Subject Headings (MeSH) and free-text keywords related to SMBG and glycemic control. Keywords included self-monitoring of blood glucose, SMBG, HbA1c, glycemic control, diabetes management, structured SMBG, and unstructured SMBG. Sensitivity was refined by applying Boolean operators such as "AND" and "OR" to the search results. To minimize publication bias, additional studies were identified by searching the reference lists of relevant systematic reviews and meta-analyses on SMBG. English-language articles from peer-reviewed journals were searched.

Eligibility Criteria

Studies were included if they involved participants diagnosed with diabetes. To ensure relevance and methodological consistency, only studies published from January 2005 onward were considered. Eligible studies specifically evaluated SMBG interventions and reported HbA1c as a primary outcome measure. Both randomized controlled trials (RCTs) and observational studies were included, provided they were published in peer-reviewed journals and the full text was accessible. Studies were excluded if they were published primarily in languages other than English, unless a translated version was available. Conference proceedings and gray literature were not included in the search strategy. Additionally, studies lacking a control group or those that did not provide sufficient HbA1c data were omitted. Research focused exclusively on gestational diabetes or prediabetes was also excluded from the review.

Data Extraction

Data extraction was conducted using a standardized template to ensure consistency and minimize errors. Study characteristics, including author, year of publication, and study design, population characteristics such as sample size and type of diabetes, and intervention details, including SMBG testing frequency and structured versus unstructured SMBG protocols, were extracted. Extracted data also included comparison groups, such as no SMBG or usual care, and primary outcomes, including HbA1c change and incidence of hypoglycemic events.

Quality and Risk of Bias Assessment

Methodological quality and risk of bias were assessed using established tools for the included studies. For RCTs, the Cochrane Risk of Bias Tool was applied to assess randomization, allocation concealment, blinding, incomplete outcome data, and selective reporting [12]. The Newcastle-Ottawa Scale (NOS) was used for observational studies, which assesses the selection of participants, comparability of groups, and ascertainment of outcomes [13]. These criteria were used to evaluate the studies and categorize them as having a low, moderate, or high risk of bias.

Statistical Analysis

Pooled effect estimates were computed through a meta-analysis using the Cochrane Collaboration Review Manager (RevMan) (Version 5.4; Copenhagen: The Cochrane Collaboration; 2020). The primary outcome was the mean difference (MD) in HbA1c levels between SMBG users and the control group, with 95% confidence intervals (CIs) calculated to determine significance. To assess heterogeneity across studies, the I² statistic was used and interpreted as follows: 0-40% indicated low heterogeneity, 41-60% indicated moderate heterogeneity, and greater than 60% indicated high heterogeneity. A random-effects model was employed to account for potential heterogeneity and variability across studies.

Results and findings

Study Selection

**Figure 1:**
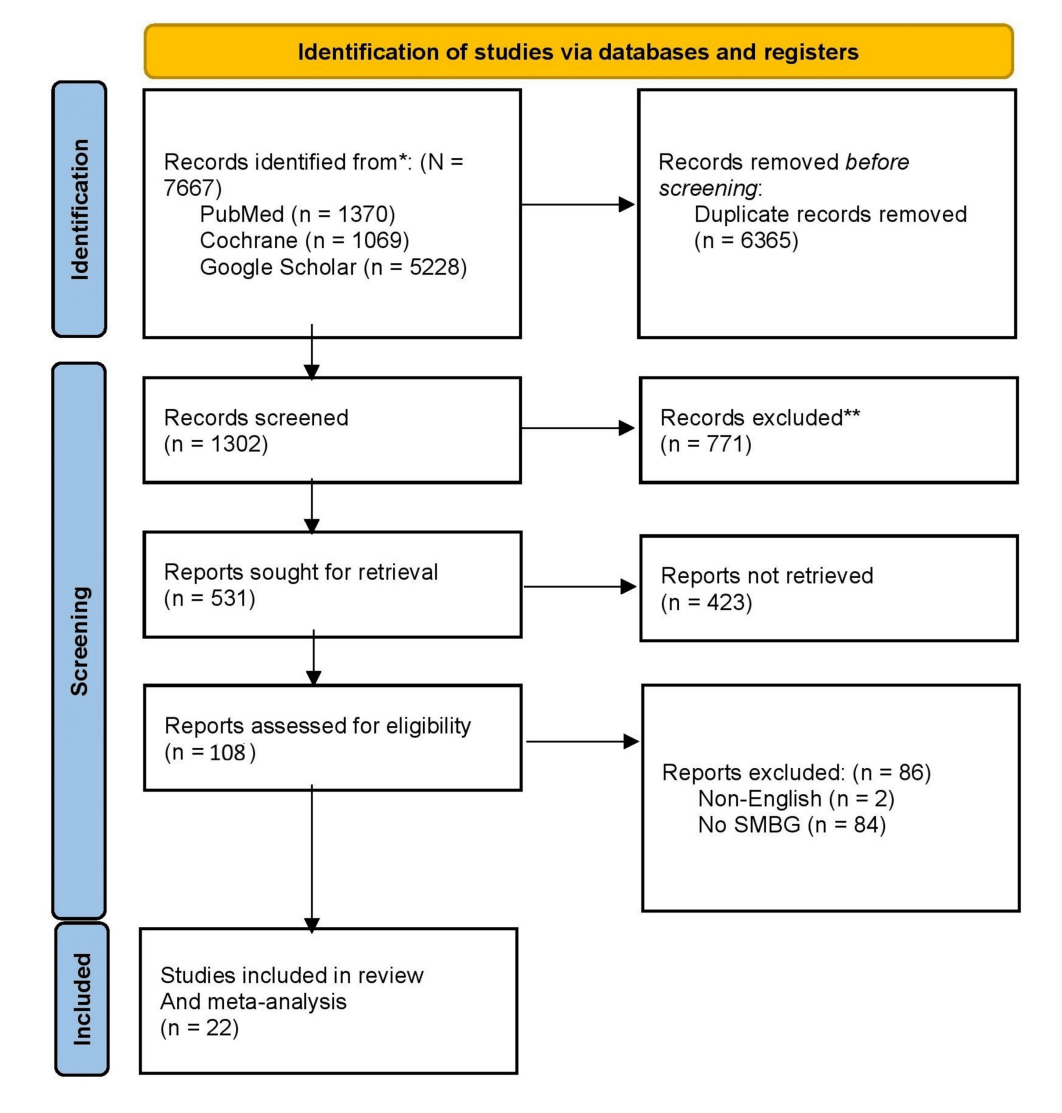
PRISMA Diagram for the Study Selection Process

A total of 7,667 records were identified through a comprehensive literature search in three primary databases: PubMed (1,370), Google Scholar (5,228), and the Cochrane Library (1,069). After removing 6365 duplicate records, 1302 unique articles were screened based on titles and abstracts. At this stage, 771 studies were excluded because they were not focused on SMBG or glycemic control. A detailed assessment of the full texts of the remaining 531 studies was then performed. Of these, 423 studies were excluded due to difficulties in retrieval or failure to report HbA1c outcomes. Two studies were non-English, and 84 did not report the influence of SMBG on HbA1c, resulting in an additional 86 exclusions. After applying all selection criteria, 22 studies were deemed eligible for inclusion in the systematic review and meta-analysis. The PRISMA diagram visually summarizes the study selection process, illustrating the progressive filtering of studies through screening, eligibility assessment, and final inclusion in the review (Figure 1).

Characteristics of Included Studies

The sample included randomized controlled trials (RCTs) and observational studies, with more than 6,500 diabetic patients pooled in total. The publication years ranged from 2005 to 2024, and the studies included populations with both Type 1 and Type 2 diabetes. The protocol for SMBG varied across studies, with either structured SMBG interventions, in which patients followed a schedule for glucose testing with clinical feedback, or unstructured SMBG, in which patient-led blood glucose monitoring was evaluated without clear guidance. Sample size, duration of intervention, and frequency of SMBG testing varied among the studies. Some studies involved only non-insulin-treated patients, while others included insulin-treated patients. The heterogeneity of SMBG approaches necessitates a separate evaluation of structured versus unstructured SMBG. Table 1 presents a summary of study characteristics, including study design, population characteristics, intervention details, and key findings.

**Table 1:** Study Characteristics RCT: randomized controlled trial, T2D: type 2 diabetes, DM: diabetes mellitus, HbA1c: glycated hemoglobin, SMBG: self-management of blood glucose, BMI: body mass index, SD: standard deviation.

Study	Year	Study Design	Population	Intervention	Comparison	Outcome Measures	Key Findings	Mean HbA1c Reduction (SMBG)	SD (SMBG)	Mean HbA1c Reduction (Control)	SD (Control)	Hypoglycemic Events (SMBG)	Hypoglycemic Events (Control)
Barnett et al. [14]	2008	RCT, Parallel-group	610 patients with T2D	SMBG with gliclazide MR	No SMBG	HbA1c reduction	SMBG group experience a reduced HbA1c by 0.25% more than no SMBG (p=0.0097)	-1.15%	1.14	-0.91%	1.29	51 events (8.7%)	66 events (7.0%)
Bosi et al. [7]	2013	RCT	1024 non-insulin-treated T2D patients	Intensive structured SMBG	Control (limited SMBG)	HbA1c reduction	Greater HbA1c reduction in SMBG group (-0.12%, p=0.013)	-0.39% (ITT)/-0.45% (PP)	2.51	-0.27% (ITT)/-0.24% (PP)	1.74	1.32 events per patient-year	0.42 events per patient-year
Davidson et al. [15]	2005	Blinded RCT	89 non-insulin-treated T2D patients	SMBG (pre/post-meal)	No SMBG	HbA1c levels	No significant difference in HbA1c	-0.8%	1.6	-0.6%	2.1	-	-
Durán et al. [16]	2010	RCT, Parallel-group	161 newly diagnosed T2D patients	SMBG-based intervention	HbA1c-based control	HbA1c reduction, BMI	Significant reduction in HbA1c (-0.5%, p<0.001) and BMI	-0.5%	0.52	-0.0%	0.85	No severe hypoglycemic episodes	No severe hypoglycemic episodes
Farmer et al. [17]	2007	RCT	453 T2D patients	SMBG (self-care education)	Usual care	HbA1c levels	No significant difference between groups	-0.15% (less intensive)/-0.18% (more intensive)	0.8/0.7	-0.01	1.02	33 patients (less intensive)/43 patients (more intensive)	14 patients
Franciosi et al. [18]	2011	RCT	62 T2D patients	SMBG + education	No SMBG	HbA1c, weight	HbA1c reduced by 1.2% in SMBG group (p=0.04)	-1.2%	0.56	-0.7%	0.53	-	-
García [19] et al.	2013	RCT	195 newly diagnosed T2D patients	SMBG-based therapy	HbA1c-based therapy	Diabetes regression rate	Higher regression rate in the SMBG group (p<0.001)	6.6±0.3 to 6.2±0.6 (SMBG)	-	6.7±0.5 to 6.8±0.6	-	-	-
Harashima et al. [20]	2013	RCT	137 T2D patients	SMBG (fingertip/palm)	No SMBG	HbA1c reduction	SMBG group experience a reduced HbA1c by 0.23% (p<0.05)	-0.2%	0.48	+0.31%	0.45	No significant difference	No significant difference
Kempf et al. [21]	2013	RCT	124 non-insulin-treated T2D patients	SMBG with lifestyle intervention	Control	HbA1c, weight	Significant HbA1c reduction in SMBG group (-0.5%, p=0.0003)	-0.5%	0.9	-0.2%	0.6	-	-
Williams et al. [22]	2020	RCT	231 non-insulin-treated T2D patients	Structured SMBG	Usual care	Glycemic variability, HbA1c	Significant improvement in glycemic variability and HbA1c (-0.7%, p<0.001)	-0.7% (7.0 mmol/mol)	-	-	-	-	-
Young et al. [23]	2017	RCT	450 non-insulin-treated T2D patients	Daily SMBG with/without feedback	No SMBG	HbA1c, HRQOL	No significant difference in HbA1c or HRQOL across groups	-0.08%	1.07	0.04%	1.12	No significant differences	No significant differences
Madeo et al. [24]	2020	Observational retrospective study	54 T2D patients	Short-term SMBG schema	Standard care	HbA1c, fasting plasma glucose	HbA1c decreased significantly in the SMBG group (p<0.001)	-0.9%	0.7	-0.8%	1.1	-	-
Malanda et al. [25]	2016	RCT	181 non-insulin-treated T2D patients	SMBG (blood/urine)	No SMBG	Diabetes distress, self-efficacy, HbA1c	No significant effect on distress, self-efficacy, or HbA1c	-0.1%	0.9	-0.2%	0.6	-	-
Muhali et al. [26]	2024	RCT	85 insulin-treated DM patients	Structured SMBG	Standard care	HbA1c, SMBG adherence	HbA1c reduced by 1.01% (p<0.001)	-1.01% (95% CI -1.39, -0.63)	-	0.18% (95% CI -0.07, 0.44)	-	-	-
Nishimura et al. [27]	2017	RCT	62 insulin-naïve T2D patients	Structured SMBG	Routine SMBG	HbA1c, weight, BP	HbA1c reduction by 0.32% in the structured SMBG group	-0.28%	0.76	-0.11%	0.57	-	-
O'Kane et al. [28]	2008	RCT	184 newly diagnosed T2D patients	SMBG	No SMBG	HbA1c, psychological indices	No significant HbA1c improvement, higher depression scores in SMBG group	-1.9%	1.84	-1.7%	1.99	-	-
Parsons et al. [8]	2019	RCT	446 non-insulin-treated T2D patients	Structured SMBG ± TeleCare	Usual care	HbA1c levels	SMBG group experience a reduced HbA1c by 0.8% vs. control (p≤0.0001)	-1.11%	1.45	-0.3%	1.41	-	-
Polonsky et al. [29]	2011	Cluster RCT	483 poorly controlled T2D patients	Structured SMBG	Usual care	HbA1c, treatment changes	SMBG group experience a reduced HbA1c by 1.2% (p=0.04)	-1.2%	1.4	-0.9%	1.5	-	-
Sodipo et al. [30]	2017	RCT	107 T2D patients	SMBG	No SMBG	HbA1c, fasting blood glucose	No statistical difference in HbA1c	-1.5%	2.36	-1.0%	2.17	-	-
Lu et al. [31]	2011	RCT	70 non-insulin-treated T2D patients	SMBG	No SMBG	HbA1c levels	SMBG group had a greater HbA1c reduction (-1.5% vs. -1.0%)	-1.5%	0.4	-1.0%	0.9	-	-
Lee et al. [32]	2017	RCT	198 non-insulin-treated T2D patients	Frequent vs. Infrequent SMBG	Standard care	HbA1c, glycemic variability	Frequent SMBG group had a greater HbA1c reduction (-2.4%)	-2.4% (Frequent)/-1.5% (Infrequent)	1.6/1.5	-1.8%	1.7	-	-
Kan et al. [33]	2017	RCT	250 diabetes patients	Structured SMBG	Routine SMBG	HbA1c reduction	The SMBG group had a significantly greater reduction in HbA1c (-1.79% to -1.91%)	-1.91% (non-insulin)/-1.79% (insulin)	1.90/1.97	-1.35% (non-insulin)/-1.05% (insulin)	1.82/1.87	-	-

Quality Appraisal

**Figure 2:**
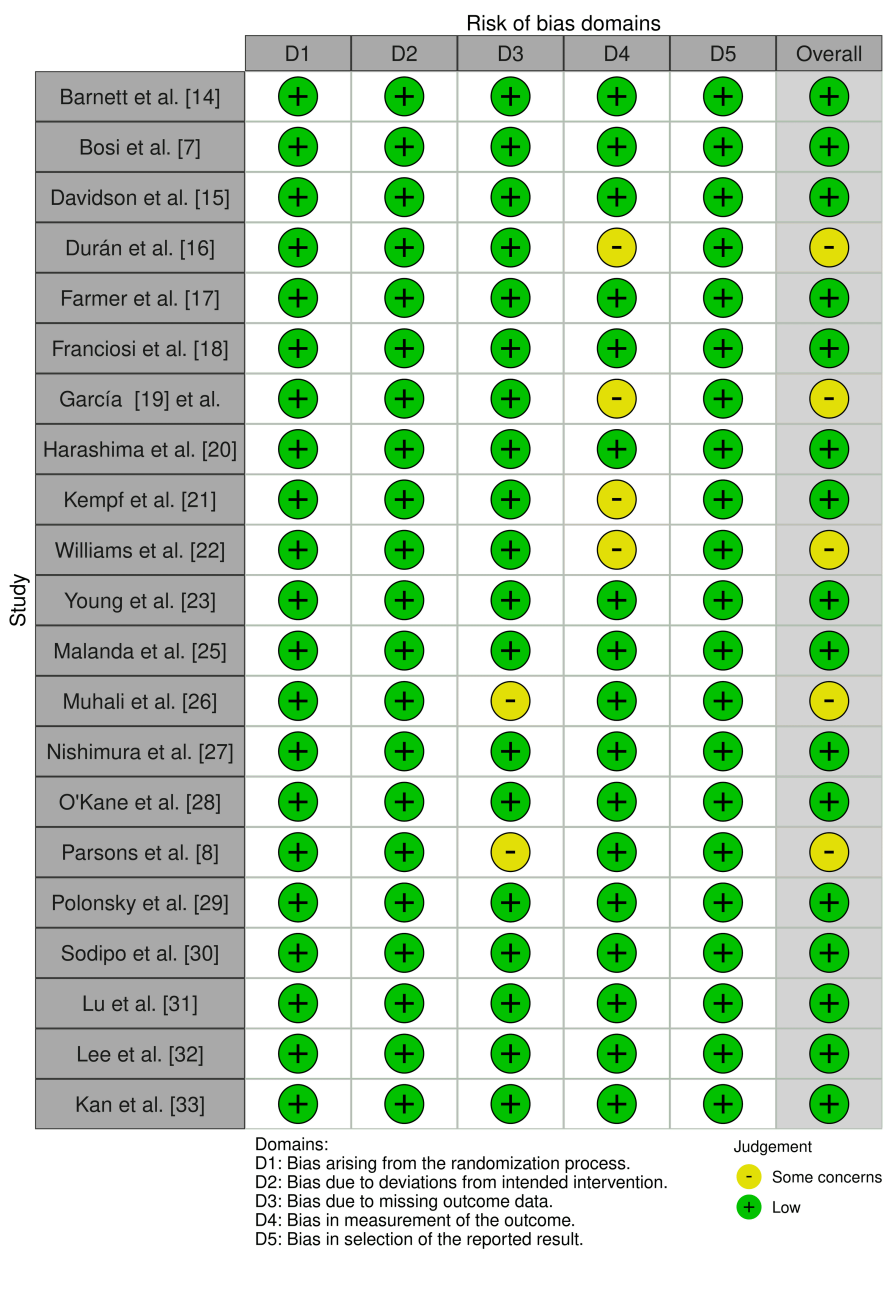
Quality Assessment of Included RCTs Using ROB 2 Data from [7,8,14-23,25-33]. RCT: randomized controlled trial, ROB 2: Risk of Bias 2.

**Figure 3:**
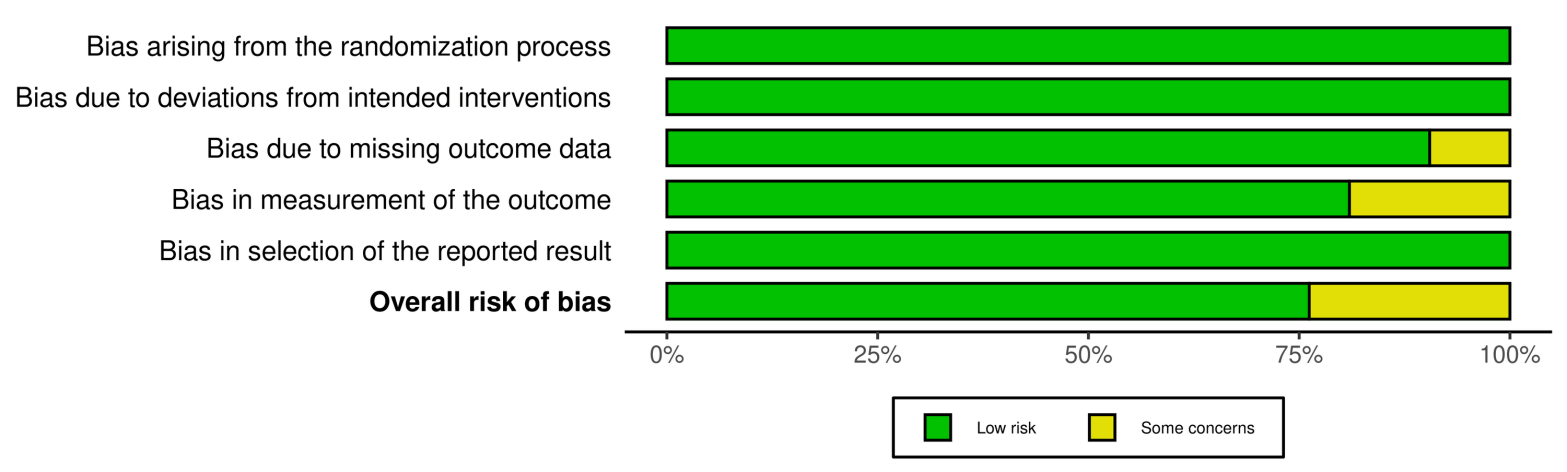
Summary Graph for Quality Assessment of Included RCTs Using ROB 2 RCT: randomized controlled trial, ROB 2: Risk of Bias 2.

Methodological quality of the included studies was estimated using the Cochrane Risk of Bias Tool for RCTs and the Newcastle-Ottawa Scale (NOS) for observational studies. The majority of the randomized controlled trials had a low to moderate risk of bias and employed strong randomization procedures, including allocation concealment and blinding procedures. The NOS score for the observational study was 7, corresponding to moderate methodological quality. The study employed stringent selection criteria that controlled for confounders but was limited by a smaller sample size and a retrospective design. These results underscore the importance of exercising caution when interpreting findings from studies with weaker controls, as such studies are often characterized by considerable variability in methodological rigor. Even with these variations, however, the quality of the included studies was sufficient to be considered reliable for understanding the impact of SMBG on glycemic control (Figures 2, 3; Table 2).

**Table 2:** Quality Appraisal of the Observational Study Using NOS NOS: Newcastle-Ottawa Scale.

Study	Selection (Max 4)	Comparability (Max 2)	Outcome (Max 3)	Total Score (Max 9)	Judgement
Madeo et al. 2020 [24]	4	1	2	7	Moderate

Meta-analysis results

**Figure 4:**
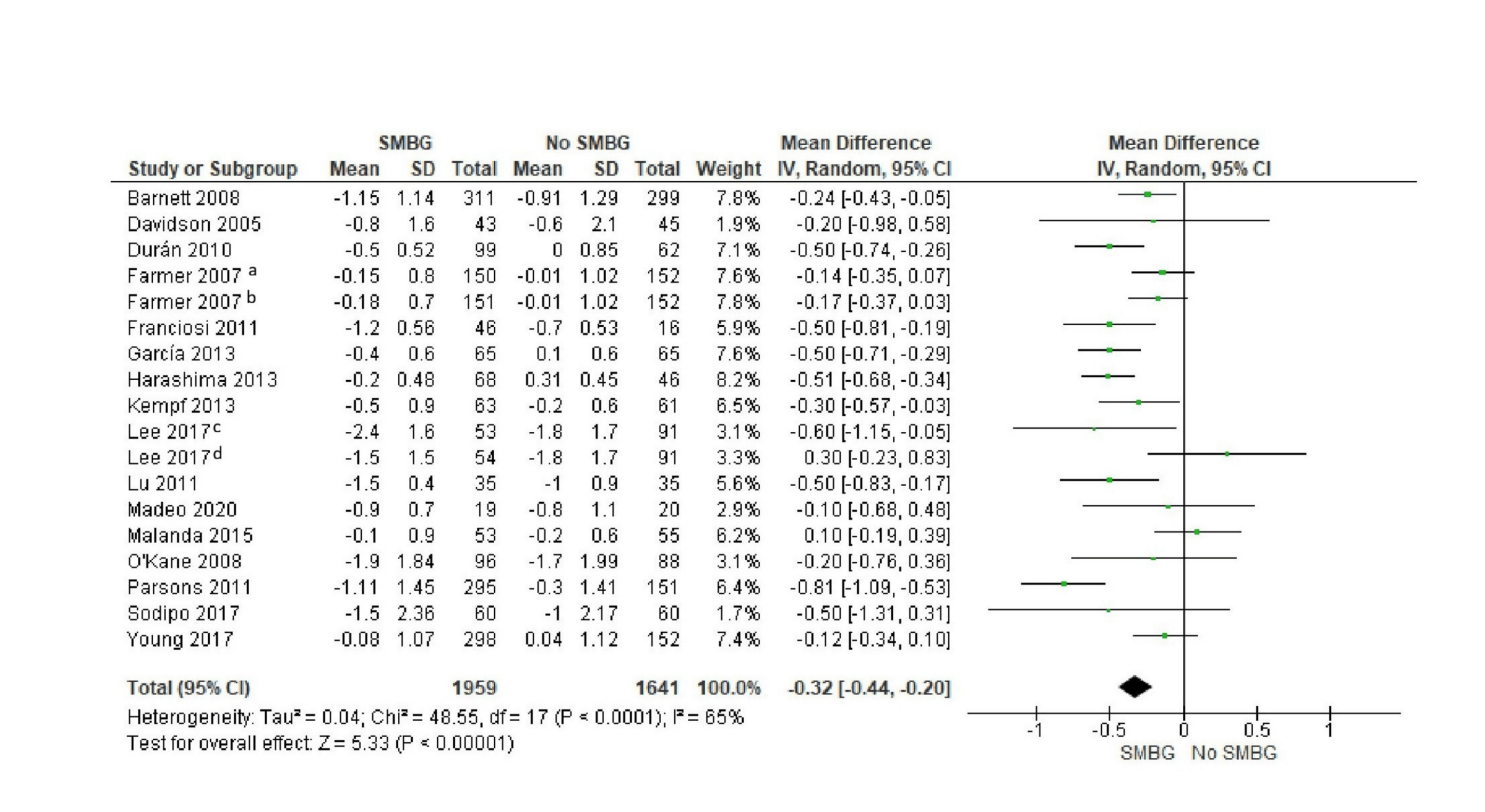
Forest Plot Showing the Mean Difference in HbA1C Between the SMBG Group and the No-SMBG Group ^a^Less intensive SMBG, ^b^more intensive SMBG, ^c^frequent SMBG, ^d^infrequent SMBG. Data from [8,14-21,23-25,28,30-32]. HbA1C: glycated haemoglobin, SMBG: self-monitoring of blood glucose.

Data from the included studies were synthesized to calculate the overall effect of SMBG on glycemic control in the meta-analysis. For the pooled results, there was a statistically significant reduction in HbA1c levels with SMBG compared to no SMBG. This result is summarized in a forest plot presenting the mean difference in HbA1c between SMBG and non-SMBG groups. Figure 4 illustrates the forest plot showing the mean difference in HbA1c between the SMBG and no SMBG groups.

The overall mean difference was -0.32 (95% CI: -0.44 to -0.20), representing a moderate but clinically meaningful improvement in glycemic control. The analysis estimated significant heterogeneity among studies (I² = 65, p < 0.0001). This heterogeneity suggests that the effect of SMBG may vary depending on the patient population, study design, intervention protocol, and adherence to SMBG guidelines.

**Figure 5:**
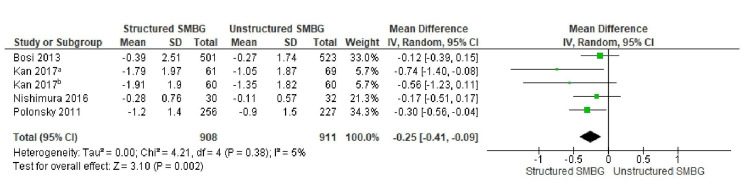
Forest Plot Showing the Mean Difference in HbA1c Between the Structured SMBG Group and the Unstructured SMBG Group. ^a^With insulin treatment, ^b^without insulin treatment. Data from [7,27,29,33]. HbA1C: glycated haemoglobin, SMBG: self-monitoring of blood glucose.

The differential impact of structured versus unstructured SMBG was examined through a subgroup analysis. There was a greater reduction in HbA1c in studies that adopted structured SMBG, in which patients tested their blood glucose in a consistent and methodical manner with guidance from clinicians, compared with studies that evaluated unstructured SMBG. A forest plot for this subgroup analysis displays the difference in HbA1c reduction between structured and unstructured SMBG interventions. Figure 5 depicts a forest plot comparing the mean difference in HbA1c between the structured SMBG and unstructured SMBG groups.

The mean difference in HbA1c reduction was -0.25% (95% CI: -0.41 to -0.09%) in favor of the structured SMBG group, which was significantly greater than that of the unstructured SMBG group. The heterogeneity in structured SMBG studies, in contrast to the overall SMBG analysis, was low, with an I² value of 5% (p = 0.38), indicating high consistency in study findings. This reinforces the need for structured monitoring protocols to optimize glycemic control.

The study data were further analyzed, which showed that the effect of SMBG was more pronounced in patients with higher initial HbA1c levels. Studies by Mannucci et al. (2018) and Ji et al. (2016) demonstrated that SMBG had the most significant effect in patients with baseline HbA1c values of 8.0% or higher. These findings suggest that SMBG may be particularly beneficial for individuals who struggle to maintain glycemic control, as it enables timely intervention and self-management adjustments. A key observation was also made regarding the role of patient education in improving the effectiveness of SMBG. Studies, including those by Polonsky et al. and Franciosi et al., which incorporated educational interventions, reported larger HbA1c reductions compared to studies that offered SMBG without structured education. Structured SMBG appears to integrate well with patient education, which may be an important factor in optimizing the clinical benefits of SMBG.

Discussion

The findings of the present study indicate that the use of SMBG is effective in lowering HbA1c levels among patients with diabetes and that structured SMBG is more effective. The meta-analysis revealed that structured SMBG was more beneficial than unstructured SMBG, yielding an overall mean difference of -0.25% in HbA1c reduction (95% CI: -0.41 to -0.09). This aligns with prior studies revealing that structured SMBG helps deliver necessary data, which may enable glucose management and enhance glycemic control [5,34]. Patients with frequent blood glucose monitoring who take positive action based on these readings enjoy superior glycemic control, as postulated by Franciosi et al. [18]. Nevertheless, non-adherence is a serious issue, as patients may fail to engage in SMBG as prescribed because of a lack of knowledge, stress, or inconvenience [25]. Considering these challenges, it appears that planning regular SMBG alongside educational interventions may represent the most effective approach.

Our study complements prior research focusing on structured SMBG in individuals with diabetes, confirming its relevance to diabetes management. In a meta-analysis of 14 clinical trials, Mannucci et al. [5] demonstrated that structured SMBG reduced HbA1c levels in patients, thereby supporting the rationale for a standardized approach to improve glycemic control. Moreover, in a more recent study by Zou et al. [34], the authors noted that increased SMBG frequency also correlated with better blood glucose control, supporting regular monitoring practices. On the other hand, some studies did not find evidence in favor of SMBG in most patients, especially those with type 2 diabetes who do not use insulin. In the study by Parsons et al. [8], there was no significant decline in HbA1c levels with SMBG among patients with non-insulin-treated type 2 diabetes. The authors concluded that SMBG may not be beneficial unless complemented by additional instructions and behavioral modifications.

According to various research studies, more frequent SMBG has been observed to improve glycemic control [6]. Those who check their blood glucose levels at least several times a day are able to notice changes in their glucose levels and adjust their behavior or medication accordingly. However, constant tracking can also have drawbacks, potentially reducing patient adherence to the regimen. Xu et al. [6] also emphasized that discussions of SMBG frequency with patients should be individualized, depending on patient characteristics, type of diabetes, and treatment plan. Several factors have been identified as obstacles to adherence, including financial charges, poor motivation, fear of pain, and inadequate understanding of the readings [10]. This becomes especially critical in the use of test strips and glucometers, as these can be expensive for low-income earners. Instructive approaches to counseling SMBG results for self-management have been found to enhance adherence and improve clinical outcomes among patients [35].

Williams et al. [22] and Mannucci et al. [5] demonstrated that structured SMBG significantly lowered HbA1c, whereas unstructured SMBG had minimal impact [9]. These observations support the recommendation to incorporate SMBG into diabetes management protocols, along with patient education and clinical interventions. The results of this review are pertinent to diabetes care in several ways. Current evidence highlights the effectiveness of structured SMBG, making it helpful for healthcare providers to develop structured SMBG guidelines and integrate them into general diabetes management.

Limitations

Variability in SMBG measurements and study populations is one of the main limitations of the included articles. Differences in diabetes type, use of structured versus unstructured SMBG, duration of disease, frequency of SMBG, and length of interventional trials were observed among the included studies. One observational study was included, which may increase bias and confounding, limit the ability to draw conclusions about causality, and potentially inflate effect estimates. Lastly, the selected publications did not consistently specify patient compliance, making it difficult to evaluate the relationship between compliance and SMBG outcomes.

## Conclusions

This systematic review and meta-analysis revealed that SMBG is effective in reducing HbA1c levels, with structured SMBG showing a greater reduction compared to unstructured SMBG. Patients who adhere to specific monitoring regimens and those who receive tailored educational interventions achieve superior glycemic management. Within clinical practice settings, patient education and structured SMBG should be incorporated into diabetes management. Potential directions for future studies include large-scale RCTs to demonstrate the long-term effectiveness of SMBG and to identify strategies to improve patient compliance. Nonetheless, it is reasonable to conclude that SMBG continues to play a significant role in diabetes management, provided that its use is optimized through well-structured protocols and consistent patient support to improve overall glycemic control and self-management practices.
